# Telemonitoring in Chronic Heart Failure: A Systematic Review

**DOI:** 10.1155/2012/410820

**Published:** 2012-06-07

**Authors:** Gregory Giamouzis, Dimos Mastrogiannis, Konstantinos Koutrakis, George Karayannis, Charalambos Parisis, Chris Rountas, Elias Adreanides, George E. Dafoulas, Panagiotis C. Stafylas, John Skoularigis, Sara Giacomelli, Zoran Olivari, Filippos Triposkiadis

**Affiliations:** ^1^Department of Cardiology, Larissa University Hospital, P.O. Box 1425, 41110 Larissa, Greece; ^2^T.E.I. of Lamia, 35100 Lamia, Greece; ^3^Department of Radiology, Larissa University Hospital, 41110 Larissa, Greece; ^4^Military General Hospital, Larissa, Greece; ^5^1st Department of Propaedeutic and Internal Medicine, Ethnikon and Kapodistriakon University School of Medical Sciences, Laikon Hospital, 11527 Athens, Greece; ^6^1st Department of Medicine, AHEPA University Hospital, 31100 Thessaloniki, Greece; ^7^Department of Cardiology, Ca' Foncello Hospital, Treviso, Italy

## Abstract

Heart failure (HF) is a growing epidemic with the annual number of hospitalizations constantly increasing over the last decades for HF as a primary or secondary diagnosis. Despite the emergence of novel therapeutic approached that can prolong life and shorten hospital stay, HF patients will be needing rehospitalization and will often have a poor prognosis. Telemonitoring is a novel diagnostic modality that has been suggested to be beneficial for HF patients. Telemonitoring is viewed as a means of recording physiological data, such as body weight, heart rate, arterial blood pressure, and electrocardiogram recordings, by portable devices and transmitting these data remotely (via a telephone line, a mobile phone or a computer) to a server where they can be stored, reviewed and analyzed by the research team. In this systematic review of all randomized clinical trials evaluating telemonitoring in chronic HF, we aim to assess whether telemonitoring provides any substantial benefit in this patient population.

## 1. Introduction

Heart failure (HF) is a growing epidemic, especially in the western world. Over the last decade, the annual number of hospitalizations has increased from 800,000 to over a million for HF as a primary diagnosis and from 2.4 to 3.6 million for HF as a primary or secondary diagnosis [1]. Approximately 50% of HF patients are rehospitalized within 6 months of discharge and with the aging of the population this trend will continue to rise [2, 3]. Understanding the epidemiology and pathophysiology of the syndrome [4], identifying the predictors and their strength of association with outcomes, and cost-effectively using the available diagnostic modalities are essential in order to devise effective preventive interventions and implement novel therapeutic approaches to curb this epidemic [5–8]. Despite, however, the emergence of novel therapeutic approached that can prolong life and shorten hospital stay [9–13], these patients will be needing rehospitalization and will often have a poor prognosis [2].

In Europe, it is estimated that at least 10 million people suffer from chronic HF [14, 15], and in the United States another 400.000–700.000 patients are diagnosed annually [16], while 1 in 9 death certificates (277,193 death) in 2007 mentioned HF [17]. The healthcare costs are equally high; in one study, it is reported that $30 billion were spent in the USA in 2007 [18].

Telemonitoring is a novel diagnostic modality that has been suggested to be beneficial for HF patients [19, 20]. Telemonitoring is viewed as a means of recording physiological data (such as body weight, heart rate, arterial blood pressure (BP) electrocardiogram (ECG) recordings, and other data) by portable devices and transmitting these data remotely (via a telephone line, a mobile phone, or a computer) to a server where they can be stored, reviewed, and analyzed by the research team.

In this systematic review of all randomized clinical trials evaluating telemonitoring in chronic HF, we aim to assess whether telemonitoring provides any substantial benefit in this patient population.

## 2. Methods

**Figure 1:**
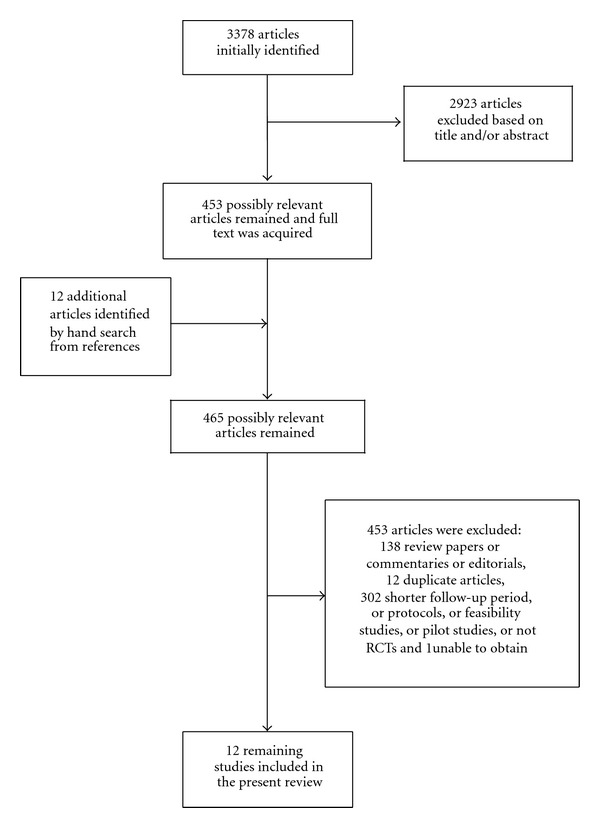
Flowchart of study search.

We searched in Medline, SpringerLink, Scopus, Cinahl, and Embase for trials that examined efficacy and efficiency of telemonitoring modalities in chronic HF patients. Keywords used in the search included: home care, telemedicine, telemetry, telemonitoring and telehealth combined with chronic heart failure. This yielded 3378, 322, 288, 130, and 48 papers respectively. The search lasted for two months and ended in November 2011. Two of the researchers read all available titles and abstracts and eliminated duplicate articles. Only randomized controlled trials were included that had a follow-up period of at least six months, clearly stated a means of telemonitoring, and were conducted in the previous ten years. We excluded feasibility or pilot studies which primarily report preliminary findings of ongoing trials, usually, in a small number of patients.  Table 1 summarizes inclusion and exclusion criteria. In Figure 1, the selection process is depicted.

## 3. Study Characteristics

We identified 12 randomized controlled trials that met our inclusion criteria (Tables 2 and 3). Sample sizes varied from 57 [33] to 710 [32] patients. The age of the participants covered a wide range from 44 [29] to 86 years [21]. In most of the studies, the functional status of the participants according to New York Heart Association's (NYHA) classification was reported (I–IV) apart from two studies [22, 30]. Two studies were multinational [24, 27], four were conducted in the USA [21, 22, 29–31], and the remaining six in Europe [21, 23, 25, 28, 32, 33].

In most of the studies, the follow-up period ranged from 6 to 12 months, while in one study participants were followed for 26 months (median value) [32]. Three studies did not clearly state left ventricular ejection fraction (LVEF) [22, 25, 30] and in all of the remaining studies, LVEF was under 40% except for one (LVEF: 35 ± 15%) [21].

Domestic telephone line was the preferred means for data transmission in most of the studies, while, in two studies cell phones were utilized [23, 32], pointing out that mobile and portable options offered by technology are being increasingly adopted in health care.

Researchers collected several physiological data. In the study by Wade and colleagues [22], body weight and BP were measured. In the study by Dendale and colleagues [21], weight, arterial blood pressure, and heart rate were monitored, while in the studies by Scherr and colleagues [23] and Giordano and colleagues [28], patients also reported the dosage of drugs taken. Goldberg et al. [29] and Soran et al. [31] recorded weight along with questions regarding HF symptoms. Cleland et al. [27] and Koehler et al. [32] monitored weight, arterial blood pressure, and ECG. In the study by Mortara et al. [24], collection of data included blood results, dyspnea score, asthenia score, edema score in addition to weight, heart rate, and systolic blood pressure. Pulse oximetry was recorded in two studies along with weight, BP, heart rate and questions regarding symptoms [25, 30]. Finally, Antonicelli et al. [33] also measured 24-hour urine output.

With regard to primary endpoints, they were similar across studies. Researchers were mostly interested in mortality (all-cause and/or cardiovascular mortality), rehospitalization, or visits to emergency department, expressed either as bed-days per year or days alive and out of hospital, and, thirdly, there were combined endpoints including the above.

## 4. Findings

In all included studies, baseline characteristics of the participants did not differ significantly between intervention and control groups. Three studies reported reduced hospitalization rates in telemonitoring groups that reached statistical significance [23, 28, 33], and another four studies also found reductions in hospitalization rates in favor of telemonitoring without, however, reaching statistical significance [21, 27, 28, 30]. In four studies there were more rehospitalizations in telemonitoring groups compared to usual care groups, but statistical significance was either not reported [25] or was not important [22, 31, 32]. Therefore, it could be argued that survival rates may occur at the expense of rehospitalization rates. However, in one study, results were mixed [24]; while the telemonitoring group in Italy had fewer hospital admissions compared to Poland and UK (3% versus 11%, *P* = 0.002), the Polish telemonitoring group had more readmissions (9% versus 3%, *P* = 0.13).

With regard to all-cause mortality, three studies reported statistically significant results that favored the telemonitoring group [21, 28, 29]. In two of these studies, mean age was relatively low (Table 2). This might implicate that younger age could be associated with better survival through improved adherence to medication plan. In the first study by Goldberg et al. [29], compliance was reported to be as high as 98.5%, while in the study by Giordano et al. [28], the authors report only that a nurse offered strategies to enhance compliance, without stating any rates of compliance. Compliance has been measured in the past and in one study by De Lusignan et al. [34] 75% of the patients recorded their weight sufficiently and blood pressure was measured at 90% of the time in the study. Medication adherence is another key-factor in this patient population. Wu et al. [35] examined World Health Organization' multidimensional adherence model (MAM) in 134 patients with a mean age of  61 ± 11  years. This model encompasses five dimensions: (1) socioeconomic factors, (2) health care system-related factors, (3) condition-related factors, (4) treatment-related factors, and (5) patient-related factors. In their multivariate analyses, worse NYHA functional class, more barriers to medication adherence (i.e., forgetting to take their medication, cost of medication), minority ethnicity, lower financial status, and lack of perceived social support, but not age nor gender, were associated with worse objectively measured medication adherence. 

In other four trials, fewer deaths were reported in the telemonitoring group in comparison to the usual care, however, these results were not statistically significant [22, 26, 31, 32]. In concordance with these positive findings, another study reported that there was no death in the telemonitoring group compared to one death in the control group [23]. In three studies all-cause mortality was not reported [24, 25, 30]. Finally, one study reported a death rate of 29% in the telemonitoring group, 27% in the telephone support group, and 45% in the usual care group at the first year (*P* = 0.032) [27].

Another issue that was investigated in three studies was the cost of hospitalization calculated per patient. One study found statistically significant reduction in the telemonitoring group compared to the usual care group (€ 843 ± 1733 versus € 1298 ± 2322, 35% reduction, *P* < 0.01) [28]. In Tompkins and Orwat's study [30], there was also a 12% reduction in the telemonitoring group (*P* = 0.14). In contrast, Dendale et al. [21] reported increased costs associated with the telemonitoring group (€ 1382 ± 3384 versus € 747 ± 2137, *P* = 0.16). 

## 5. Discussion

Since an aspect of medicine is the continuing attempt to provide better care to people and HF patients in particular, it is worth trying to identify the way and means to improve their quality of life through the best available evidence-based knowledge. There are several meta-analyses in the literature that offer an interpretation of findings after a statistical process of different trials. These results are based on solid mathematical procedures, offered by a computer program. In our opinion, there will always be a degree of error involved, inherent in all human processes. That is, despite the effort of all esteemed researchers, there will still be discrepancies in study designs which may render them not absolutely comparable. There are inclusion and exclusion criteria differences among studies, functional status differences, outcome measure discrepancies, and so on.

Currently available trial results may seem rather ambiguous and confusing. Nevertheless, it appears that the above-presented randomized controlled trials tend to be in favor of telemonitoring. It could be argued that in some studies sample sizes were small and thus underpowered to detect significant associations. Importantly, however, an improved quality of life—a soft end-point gaining more and more clinical significance—has been reported in all studies, whereas telemonitoring was highly acceptable by chronic HF patients.

Key components that patients with HF encounter through their contact with healthcare services should be identified in order to design larger scale studies that could test their value. Small-sized trials may provide some insight; however, this should always be verified by larger trials. In the field of telemonitoring, protocols should be clear beforehand. It may be of great importance in case participants are asked to monitor their status daily or every other day. Patient education is also important and documentation of learning goals and results should be provided, a task that can be undertaken by experienced nurses.

Another urgent need is the identification of patients that would actually be benefited by such interventions. Since the resources are getting scarce and in a time when cutbacks and cost reductions are getting bigger, sustainability of telemonitoring approaches seems difficult. Consequently, a key factor that will influence the future implementation of telemonitoring strategies is the availability of human and economic resources.

## Figures and Tables

**Table 1:** Inclusion and exclusion criteria.

*Inclusion criteria*	
Randomized controlled trials (RCT)	
Trials conducted in the previous ten years	
At least one device that measures physiological data provided by the researchers for home use	
Intended (per protocol) follow-up period of at least 6 months	
*Exclusion criteria*	
Papers that published protocols	
Papers that published feasibility data	
Papers that published pilot studies	
Review papers	
Papers not in English	

**Table 2:** Study characteristics and participants' data.

Reference	Sample size	Age^†^	E.F.	Follow-up period	Transmission modality	NYHA^a^ class	Study design	Place
(1) Dendale et al. [21]	160	76 ± 10	35 ± 15%	6 m	Cell phone	>II	RCT^b^	7 hospitals in Belgium
(2) Wade et al. [22]	316	78.1	Not reported	6 m	Internet link	Not reported	RCT^b^	New York, New Jersey, Pennsylvania residents
(3) Scherr et al. [23]	120	66 (median, IQR^c^ 62–72)	<38%	6 m	Mobile phone	II–IV	RCT^b^	Austria
(4) Mortara et al. [24]	461	60 ± 11	29 ± 7	12 m	Telephone line	II–IV (2.4 ± 0.6)	RCT^b^	11 centers in Italy, UK, and Poland
(5) Dar et al. [25]	182	72 (Mean) SD^d^: 12	Not reported	6 m	Telephone line	II–IV	RCT^b^	3 acute hospitals in northwest London
(6) Antonicelli et al. [26]	57	78 (Mean) SD^d^: 7		12 m	Telephone line	II–IV	RCT^b^	Italy
(7) Cleland et al. [27]	426	67 (Mean) SD^d^: 12	<40%	8 m	Telephone line	I–IV	RCT^b^	16 hospitals in Germany, UK and The Netherlands
(8) Giordano et al. [28]	460	57 ± 10	<40%	12 m	Telephone line	II–IV	RCT^b^	5 cardiovascular rehabilitation departments in Italy
(9) Goldberg et al. [29]	280	59 ± 15	<35%	6 m	Telephone line	III-IV	RCT^b^	16 heart failure centres in the USA
(10) Tompkins and Orwat [30]	390	76.1 (SD^d^: 8.1)	Not reported	6 m	Telephone line	Not reported	RCT^b^	Arizona, USA
(11) Soran et al. [31]	315	76 ± 7	23 ± 9%	6 m	Telephone line	II-III	RCT^b^	3 cites in Pittsburg, Cleveland, and Miami, USA
(12) Koehler et al. [32]	710	66.9 ± 10.7	≤35%	26 m (median)	Cell phone	II-III	RCT^b^	165 practices in Germany

^†^Age is reported in years as a mean value unless otherwise stated.

^a^NYHA: New York Heart Association, ^b^RCT: randomized controlled trial, ^c^IQR: interquartile range, ^d^SD: standard deviation.

**Table 3:** Data measured, hospitalization rates and costs, primary endpoints, and all-cause mortality of trials.

	Physiological parameters measured	Cost of hospitalization per patient (telemonitoring {TM} group versus control group)	All-cause mortality (TM group versus control group)	Hospitalization rates or percentages (%) in TM group versus control group	Primary endpoints
Dendale et al. [21]	W^a^, BP^b^, HR^c^	1382€ ± 3384 versus 747€ ± 2137 (*P* = 0.16)	5% versus 17.5% (*P* = 0.01)	0.24 versus 0.42 (*P* = 0.06)	All-cause mortality
Wade et al. [22]	W^a^, BP^b^	Not reported	3.7 versus 3.9 (*P* = 0.96)	34.8% versus 32.2% (*P* = 0.53)	Hospital admission, emergency department visit or death
Scherr et al. [23]	W^a^, BP^b^, HR^c^, D^d^	Not reported	0 in intervention group, 1 in control group	54% RR^e^ reduction, Confidence Interval 7 to 79%, (*P* = 0.04) in favor of intervention group	Cardiovascular mortality or rehospitalization for worsening HF^f^
Mortara et al. [24]	W^a^, HR^c^, SAP^g^, DS^h^, AS^i^, OS^j^, changes in therapy, blood results	Not reported	Not reported separately	Italy versus Poland and UK: 3 versus 11% (*P* = 0.002). Poland: 9 versus 3% (*P* = 0.13)	Bed-days/year, Death+hospitalization due to HF^f^
Dar et al. [25]	W^a^, BP^b^, HR^c^, PO^k^, questions about symptoms	Not reported	Not reported	36% versus 25%	Days alive and out of hospital, all-cause hospitalizations
Antonicelli et al. [26]	W^a^, BP^b^, HR^c^, 24 h urine output, weekly ECG	Not reported	3 cases versus 5 cases, non significant	9 cases versus 25 cases (*P* < 0.05)	Rate of mortality and hospitalization
Cleland et al. [27]	W^a^, BP^b^, HR^c^, ECG	Not reported	29% versus 27% (telephone support-{TS} group) versus 45% at 1st year (*P* = 0.032)	47% (TM) versus 49% (TS) versus 54%	Days lost due to death or all cause hospitalization
Giordano et al. [28]	W^a^, BP^b^, ECG, drug dosage	843€ ± 1733 versus 1298€ ± 2322 (−35%, *P* < 0.01)	9% versus 14%	24% versus 36% (RR = 0.57, CI: 0.38 to 0.82; *P* = 0.01)	Unplanned hospital admission for cardiovascular reason
Goldberg et al. [29]	W^a^, symptom questions	Not reported	8% versus 18.4% (*P* < 0.003)	0.19 ± 0.46 versus 0.20 ± 0.30 (*P* = 0.28)	180-day hospital readmission rate
Tompkins and Orwat[30]	W^a^, BP^b^, HR^c^, PO^k^, symptom questions	12% reduction of total cost in TM group (*P* = 0.14)	Not reported	Lower hospital admissions in TM group, incidence rate ratio = 0.87	Inpatient hospital utilization
Soran et al. [31]	W^a^, symptom questions	Not reported	7.0% versus 11.2% (*P* = 0.24)	46.8% versus 42.5% (*P* = 0.44)	Cardiovascular death or rehospitalization for heart failure
Koehler et al. [32]	W^a^, BP^b^, ECG,	Not reported	54 cases versus 55 cases (hazard ratio 0.97, CI = 0.67 to 1.41, *P* = 0.87)	486 events versus 394 events (hazard ratio 1.12, CI = 0.91 to 1.37, *P* = 0.29)	Death from any cause

^a^W: weight, ^b^BP: arterial blood pressure, ^c^HR: heart rate, ^d^D: dosage of heart failure medication, ^e^RR: relative risk, ^f^HF: heart failure ^g^SAP: systolic arterial pressure, ^h^DS: dyspnoea score, ^i^AS: asthenia score, ^j^OS: oedema score, ^k^PO: pulse oximetry.
